# A Prognostic Gene Signature Expressed in Primary Cutaneous Melanoma: Synergism With Conventional Staging

**DOI:** 10.1093/jncics/pky032

**Published:** 2018-07-23

**Authors:** Georg Brunner, Achim Heinecke, Thomas M Falk, Beyhan Ertas, Norbert Blödorn-Schlicht, Hans-Joachim Schulze, Ludwig Suter, Jens Atzpodien, Carola Berking

**Affiliations:** 1 Department of Cancer Research; 2 Department of Dermatology, Skin Cancer Center Hornheide, Muenster, Germany; 3 Department of Biometry and Clinical Research, Westphalian Wilhelms University, Muenster, Germany; 4 Dermatologikum Hamburg, Hamburg, Germany; 5 Department of Medical Oncology, Niels Stensen Clinics, Osnabrück, Germany; 6 Department of Dermatology, University Hospital, Munich, Germany

## Abstract

**Background:**

Current clinico-pathological American Joint Committee on Cancer (AJCC) staging of primary cutaneous melanoma is limited in its ability to determine clinical outcome, and complementary biomarkers are not available for routine prognostic assessment. We therefore adapted a gene signature, previously identified in fresh-frozen (FF) melanomas and adjacent stroma, to formalin-fixed paraffin-embedded (FFPE) melanomas. The aim was to develop a gene expression profiling (GEP) score to define patient survival probability at the time of first diagnosis.

**Methods:**

Expression of 11 FF melanoma signature genes was quantified by reverse transcription polymerase chain reaction in an FFPE melanoma training cohort (n = 125), corresponding to the combined FF melanoma training and validation cohorts. The resulting GEP score was validated technically and clinically in an independent FFPE melanoma cohort (n = 211). All statistical tests were two-sided.

**Results:**

We identified a prognostic eight-gene signature in the tumor area (tumor and adjacent tissue) of AJCC stage I–III melanomas. A signature-based GEP score correlated with melanoma-specific survival (MSS; Kaplan-Meier analysis: *P* < .0001) was independent of tumor stage (multivariable regression analysis: *P* = .0032) and stroma content (<5%–90%) and complemented conventional AJCC staging (receiver operating characteristic curve analysis: area under the curve = 0.91). In the clinical validation cohort, the GEP score remained statistically significant (*P* = .0131) in a multivariable analysis accounting for conventional staging. The GEP score was technically robust (reproducibility: 93%; n = 84) and clinically useful, as a binary as well as a continuous score, in predicting stage-specific patient MSS.

**Conclusions:**

The GEP score is a clinically significant prognostic tool, contributes additional information regarding the MSS of melanoma patients, and complements conventional staging.

Melanoma is one of the most aggressive types of skin cancer, accounting for 75% of skin cancer–related mortality (1). A characteristic feature of melanoma is the ability to metastasize at early stages of tumor progression (2). For decades, metastatic melanoma (American Joint Committee on Cancer [AJCC] stage IV) has been difficult to treat with conventional therapies, resulting in poor median survival of six to 12 months (1). Within the past years, rapidly evolving immunological and targeted therapies have extended the life expectancy of patients with advanced melanoma (1,3,4). These novel treatment options are currently finding entry into the adjuvant therapeutic setting (5), starting with recent US Food and Drug Administration approval of the CTLA4 inhibitor ipilimumab and the PD1 inhibitor nivolumab for adjuvant treatment of AJCC stage III patients.

However, broad application in the adjuvant treatment of clinically tumor-free patients is hampered by the considerable side effects and high costs associated with these promising new therapies. Future treatment strategies will, therefore, require precise identification of patients at high risk of relapse. However, conventional AJCC staging of primary melanoma, based on histopathological and clinical criteria, is limited in its ability to provide a definite prognosis for all patients. Therefore, new prognostic biomarkers complementing conventional staging are required to accurately identify truly high-risk patients in need of adjuvant therapy.

## Methods

### Subjects and Tissue Specimens

Following written informed consent of the patients, tissue samples were used for this study. Procedures were approved by the local Ethics Committee of the University of Münster, Germany. Previously, primary melanomas across AJCC 2009 stages IA–IIIC had been prospectively recruited for our FF tissue study by unbiased chronological collection between 1983 and 2006 at the University Hospital Munich and at the Skin Cancer Center Hornheide in Münster, both in Germany (6). In the present study, FFPE melanoma tissue of the patients in our previous FF training and validation study cohorts (n = 135) was used as the training cohort. To ensure that tissue blocks were representative of the tumor, samples were reviewed, following hematoxylin and eosin staining, to confirm diagnosis and assess tumor thickness. Only samples with a maximal thickness of 50% or more of the diagnosed Breslow thickness were included in the study. Out of the 135 melanomas, 125 FFPE tissue blocks met the inclusion criteria and yielded RNA of sufficient quantity and quality (Table 1). Median follow-up was 96 (3–273) months, and the clinical end point was patient melanoma-specific survival (MSS). Follow-up time for long-term survivors was at least five years.

**Table 1.** Clinical and histological characteristics of the training and validation cohorts*

Characteristics	Training cohort (n = 125)	Validation cohort (n = 211)
Median age (range), y	59 (19–88)	58 (23–90)
Sex, No.		
Male	67	108
Female	58	103
Breslow thickness, median (range), mm	1.9 (0.22–34)	2.84 (0.21–24)
≤1 mm, No.	41	27
1.01–2 mm, No.	24	44
2.01–4 mm, No.	29	68
>4 mm, No.	31	72
Ulceration, No.		
Absent	91	99
Present	34	112
AJCC stage at diagnosis, No.
IA	37	14
IB	17	29
IIA	18	25
IIB	14	32
IIC	9	30
IIIA	9	24
IIIB	11	32
IIIC	10	25
Median follow-up (range), mo	96 (3–273)	64 (2–316)

AJCC = American Joint Committee on Cancer, seventh edition, of the Cancer Staging Manual.

The validation cohort comprised 211 independent melanomas recruited between 1979 and 2008 (Table 1). In contrast to the prospectively sampled training cohort, the validation cohort was retrospectively selected. The rationale for sample selection was fourfold: 1) to avoid high prevalence of potentially easy-to-prognosticate stage I melanomas, 2) to achieve equal distribution across all eight relevant AJCC 2009 substages, IA–IIIC (approximately 25–30 samples/stage; except for stage IA, due to the scarcity of high-risk melanomas), 3) to balance substages regarding the proportions of short-term (MSS of five years or less) vs long-term survivors (MSS of five or more years), and 4) to perform validation in a cohort that was difficult to prognosticate, comprising deliberately selected patients with five-year survival outcomes that differed, in 40% of the cases, from those expected according to binary AJCC staging (ie, patients in stages I, IIA, IIB, IIIA with melanoma-specific death within five years or patients in stages IIC, IIIB, IIIC with five or more years of survival) (Table 3). Median clinical follow-up was 66 (2–316) months, and the clinical end point was MSS. Follow-up time for long-term survivors was at least five years.

**Table 3.** Multivariable regression analysis of the association with MSS (GEP score vs AJCC 2009 stage)

Variable	Range	Dichotomization	Hazard ratio (95% CI)	*P*
Training cohort (n = 125)				
GEP score	–0.84 to 3.55	<1.3 to ≥1.3*	3.09 (1.46 to 6.53)	.0032
AJCC 2009 stage	IA to IIIC	I, IIA, IIB, IIIA–IIC, IIIB, IIIC	5.77 (2.75 to 12.10)	<.0001
Validation cohort (n = 211)				
GEP score	–0.21 to 3.38	<1.3 to ≥1.3*	1.73 (1.12 to 2.67)	.0131
AJCC 2009 stage	IA to IIIC	I, IIA, IIB, IIIA–IIC, IIIB, IIIC	1.53 (0.99 to 2.35)	.0506

* Cutoff value used for dichotomization. AJCC = American Joint Committee on Cancer; CI = confidence interval; GEP = gene expression profiling.

### Gene Expression Analysis

Total RNA was prepared from whole FFPE tissue sections (tumor and adjacent tissue, 3–5-µm thick) of a representative tissue block (≥50% of Breslow thickness) by deparaffinization, mechanical homogenization, and use of RNeasy FFPE Kits (Qiagen, Hilden, Germany). The rationale to include adjacent tissue in the analysis, that is, using whole tissue sections as opposed to macro-dissected tumor tissue, was the biological significance of the stroma (particularly the tumor–stroma interface) in regulating tumor growth and progression. RNA was quantified and quality-controlled by spectrophotometry and reverse transcription polymerase chain reaction (RT-PCR) of three housekeeping genes (*GAPDH*, *GUSB*, and *BPNT1*). Total RNA was reverse-transcribed using High Capacity Reverse Transcriptase Kits (Applied Biosystems, Foster City, CA). Total Human Reference RNA (Agilent Technologies, Santa Clara, CA) was used as a standard. cDNA was preamplified (14 cycles) using TaqMan PreAmp Master Mix Kits (Applied Biosystems) and pooled TaqMan assays of signature and housekeeping genes.

Gene expression was quantified by TaqMan-based real-time PCR (Applied Biosystems) (6,7) of preamplified cDNA. Interassay variability between PCR runs was corrected for by data normalization (Δ cycle threshold [CT] method) using Human Reference RNA as an internal standard (reference: mean CT_ref_ of 20.0, of all 11 signature and housekeeping genes in reference RNA, determined across 20 RT-PCR runs). Interassay variability, however, proved to be almost negligible. Intraassay variability, due to varying sample RNA quality, was corrected for by data normalization (ΔCT method) using housekeeping genes as internal standards (reference: mean CT_hkg_ of 23.5, of the above three housekeeping genes, determined across 125 training samples). As expected, intersample variability proved to be considerable. Samples with an average CT_hkg_ greater than 28.5 were excluded from analysis because of low RNA quality.

### Development, Statistical Evaluation, and Validation of a Prognostic Gene Signature

The strategy for analyzing gene expression data in the training cohort was analogous to that developed previously for FF melanomas (6). Because of the wide data range (more than six logs of mRNA copy numbers) and the frequently observed asymmetry of gene expression profiles, CT values for each gene were dichotomized. Despite a potential loss of statistical power, this allowed for the identification of a robust gene signature by minimizing the impact of experimental and/or biological variability (8), for example, due to anatomical tumor localization, to spatial variability within the tumor, or to the proportion of stroma in the tissue sample. Dichotomization was based on the ratio of high-risk (MSS of less than five years; 30% of patients) vs low-risk melanomas (MSS of five or more years; 70% of patients) in the cohort. This was based on the assumption that the distribution of high-score vs low-score data should correspond to the ratio of high-risk vs low-risk patients in the training cohort.

The prognostic significance of the association of gene expression with MSS was evaluated by univariate Cox regression analysis, either as risk genes (expression inversely correlated with MSS) or as protective genes (expression correlated with MSS). To develop an algorithm to calculate a gene expression profiling (GEP) score correlating with clinical outcome, expression of prognostically significant signature genes was coded as follows. Low risk (coded as 0): CT lower than cutoff for risk genes or greater than or equal to cutoff for protective genes; high risk (coded as 1): CT greater than or equal to cutoff for risk genes or lower than cutoff for protective genes. For the protective palmoplantar keratin *KRT9*, a second cutoff was introduced at high expression levels (CT < second cutoff = high risk [coded as 1]) to account for the poor prognosis of acral melanomas (9). The GEP score was calculated as the sum of the coded values of the signature genes multiplied by the regression coefficient of each gene, obtained from multivariable Cox regression analysis. The GEP score profile of the training cohort was either dichotomized as described above or used as a continuous parameter.

When used as binary parameters, prognostic misclassification was defined as follows. 1) GEP score: Low score of less than 1.3 and patient survival of less than five years, high score of 1.3 or greater and patient survival of five or more years; 2) AJCC stage: stages I, IIA, IIB, IIIA and patient survival of less than five years, stages IIC, IIIB, IIIC and patient survival of five or more years.

The prognostic significance of the association of the GEP score with MSS was evaluated by Kaplan-Meier analysis, by the log-rank test, and by multivariable Cox regression analysis. The latter comprised GEP score, AJCC stage, age, and sex. All *P* values were two-sided. In addition, the prognostic performance of the GEP score in predicting five-year MSS was documented by receiver operating characteristic (ROC) analysis.

## Results

### Identification of a Prognostic Signature: Association With Patient Melanoma-Specific Survival

To establish a prognostic gene signature applicable to FFPE primary melanomas, we analyzed expression of 11 candidate genes derived from our previous whole-transcriptome analysis of FF melanomas (6) in the FFPE melanoma training cohort. In univariate Cox regression analysis, expression of eight of the 11 genes was statistically significantly associated with MSS (Table 2). This eight-gene signature comprised seven protective genes (high expression in low-risk melanomas): keratin 9 (*KRT9*), dermcidin (*DCD*), prolactin-induced protein (*PIP*), secretoglobin family 1D member 2 (*SCGB1D2*), secretoglobin family 2A member 2 (*SCGB2A2*), collagen alpha6(VI) (*COL6A6*), guanylate binding protein 4 (*GBP4*), and one risk gene (high expression in high-risk melanomas): kelch-like family member 41 (*KLHL41*).

**Table 2.** Association of the expression of 11 candidate genes with patient MSS (training cohort, n = 125)

Gene (prognosis)*	UniGene number	CT cutoff†	*P*‡ (FFPE)	*P*‡,§ (FF)
*KRT9* (protective)	Hs.654569	24.60/31.05	.0001	.001
*DCD* (protective)	Hs.350570	22.43	.0001	.004
*PIP* (protective)	Hs.99949	26.58	.0001	.007
*SCGB1D2* (protective)	Hs.204096	27.72	.0001	.024
*SCGB2A2* (protective)	Hs.46452	28.76	.003	.025
*COL6A6* (protective)	Hs.591282	28.87	.004	.057
*GBP4* (protective)	Hs.409925	26.64	.012	n.s.
*KLHL41* (risk)	Hs.50550	28.16	.031	.003
*ECRG2* (risk)	Hs.244569	29.97	n.s.	.006
*HES6* (risk)	Hs.42949	29.23	n.s.	.096
*MUC7* (protective)	Hs.631946	30.58	n.s.	n.s.

* Expression of protective genes was correlated with MSS, and expression of risk genes was inversely correlated with MSS. CT = cycle threshold; FF = fresh-frozen; FFPE = formalin-fixed paraffin-embedded; MSS = melanoma-specific survival; n.s. = statistically nonsignificant.

† CT cutoff value used for dichotomization.

‡ *P* values for the association of gene expression with MSS were determined by univariate Cox regression analysis.

§ Data taken from Brunner et al. (6).

Both the eight-gene FFPE melanoma signature and our previous nine-gene FF melanoma signature were derived from the same set of the above 11 candidate genes. When comparing the FFPE with the FF melanoma signature, two candidate genes were missing (esophageal cancer–related gene 2 [*ECRG2*] and hairy and enhancer of split 6 [Drosophila] [*HES6*]) and guanylate binding protein–4 (*GBP4*) was included. Intriguingly, the prognostic power of most signature genes (except of *KLHL41*) was increased by more than 10-fold in FFPE melanomas when compared with their FF counterparts (Table 2). This is most likely due to the more rigorous data normalization required for FFPE gene expression analysis.

### Prognostic Performance of a Signature-Based GEP Score

Based on the eight-gene FFPE signature, a GEP score was calculated as the sum of the coded expression data of the genes (low risk = 0, high risk = 1; see the “Methods”), weighted with the regression coefficients obtained from Cox regression analysis:

Gene  expression profiling (GEP) score= 0.94 x KRT9 + 0.70 x DCD - 0.49 x PIP + 1.58 x SCGB1D2 - 0.63 x SCGB2A2+ 0.33 x COL6A6 + 0.67 x GBP4 – 0.21 x KLHL41

**Figure 1.**
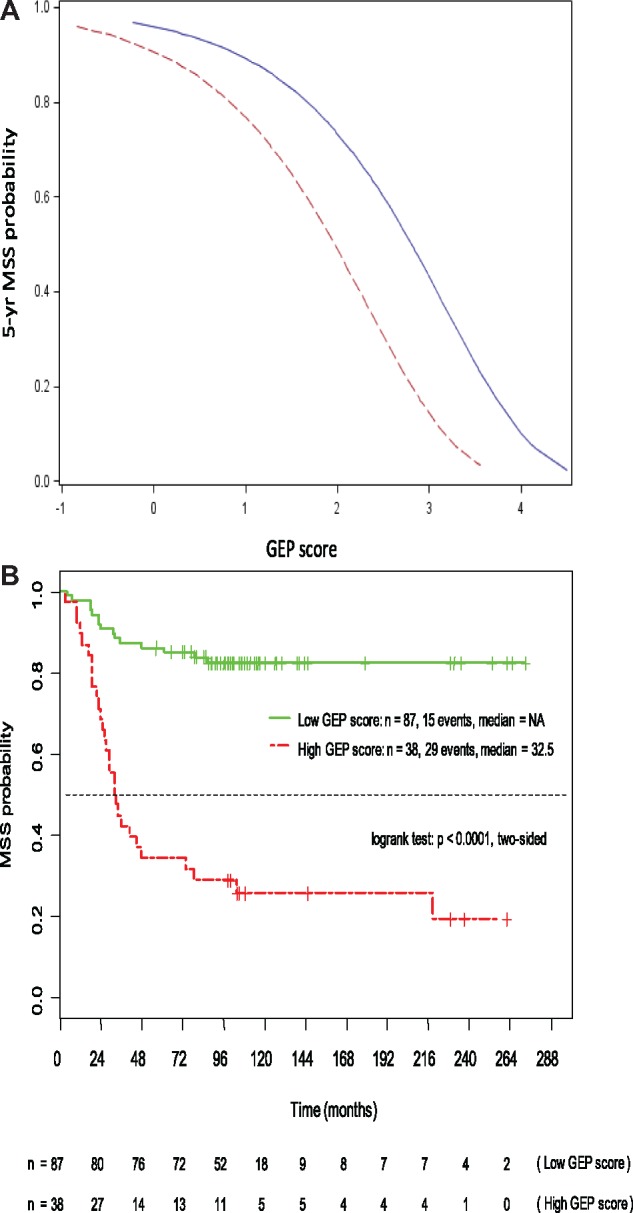
Gene expression profiling (GEP) score–dependent patient melanoma-specific survival (MSS) probability in the overlapping fresh-frozen (FF) and formalin-fixed paraffin-embedded (FFPE) training cohorts. **A)** Regression analysis according to the continuous GEP score (**red**: eight-gene FFPE score, n = 125; **blue**: nine-gene FF score, n = 91). **B)** Kaplan-Meier estimates according to the dichotomized FFPE GEP score (**green**: GEP score <1.3, n = 87; **red**: GEP score ≥1.3, n = 38). Median follow-up was 96 (3–273) months; two-sided log-rank test: *P *<* *0.0001. GEP = gene expression profiling; MSS = melanoma-specific survival.

The GEP score profile of the FFPE training cohort ranged from –0.84 to 3.55, and association of the continuous GEP score with MSS probability was similar to that of the FF-GEP score (Figure 1A, red vs blue line). Following dichotomization of the FFPE-GEP score profile (<1.3, low risk; ≥1.3, high risk), association with MSS was evaluated by Kaplan-Meier analysis (Figure 1B). The GEP score discriminated statistically significantly between short-term and long-term survivors (across all relevant AJCC stages, IA–IIIC; *P* < .0001, log-rank test).

**Figure 2.**
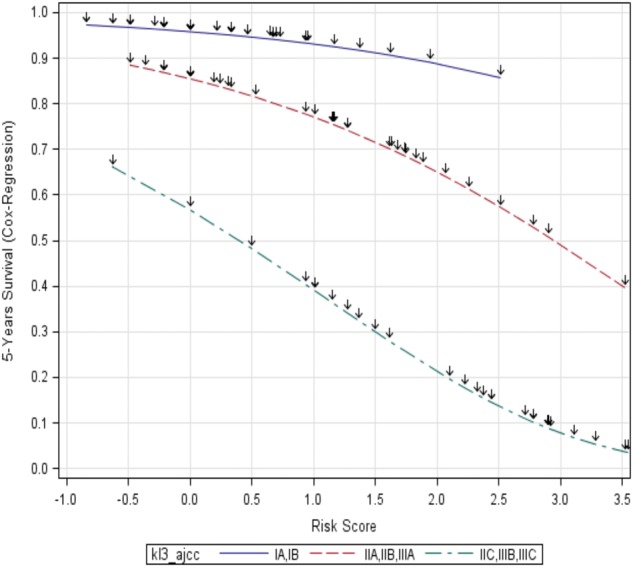
Cox regression analysis of five-year melanoma-specific survival (MSS) probability according to the continuous gene expression profiling (GEP) score. Three subgroups of the training cohort are shown; **blue**: stage I = low risk, n = 54; **red**: stages IIA/IIB/IIIA = intermediate risk, n = 41; **green**: stages IIC/IIIB/IIIC = high risk, n = 30). GEP score distribution within the subgroups is indicated by **arrows**. Median follow-up was 96 (3–273) months.

To evaluate whether the GEP score contributes independent prognostic information in addition to conventional staging, multivariable Cox regression analysis was performed, comprising GEP score, AJCC stage, Clark level, age, and sex. Only GEP score (hazard ratio [HR] = 3.09, *P* = .0032) and AJCC stage (HR = 5.77, *P* < .0001) were statistically significantly associated with MSS (Table 3). Thus, the GEP score was statistically independent and complemented conventional AJCC staging.

Dichotomizing the GEP score is associated with a loss of information as compared with using the GEP score as a continuous variable. We therefore analyzed whether the continuous GEP score could also contribute additional prognostic information in the AJCC-stage based risk groups, stage I (low risk, five-year MSS probability 96% [10]), stages IIA/IIB/IIIA (intermediate risk, average MSS probability 77%), and stages IIC/IIIB/IIIC (high risk, MSS probability 52%). The continuous GEP score complemented AJCC staging by specifying MSS probability in each risk group across a relatively large range (low risk: 86%–98%; intermediate risk: 40%–89%; high risk: 4%–67%) (Figure 2).

Synergistic performance of the continuous GEP score and AJCC staging was further illustrated by ROC analysis (Table 4). The highest prognostic precision was achieved by combining AJCC staging and GEP score (area under the curve value = 0.91 in the training cohort).

**Table 4.** ROC analysis of the prediction of five-year MSS by continuous GEP score, AJCC 2009 staging, or a combination of both

AUC value	Training cohort (n = 125)	Validation cohort (n = 211)
GEP score (–0.84 to 3.55)	0.85	0.65
AJCC stage (I, II, III)*	0.87	0.60
AJCC stage + GEP	0.91	0.66

* Stage III was used as a reference. AJCC = American Joint Committee on Cancer; AUC = area under the curve; GEP = gene expression profiling; MSS = melanoma-specific survival; ROC = receiver operating characteristic curve.

### Technical Validation of the GEP Score

To evaluate the interassay variability of the GEP score, 84 FFPE melanomas of the training cohort were re-analyzed in four different laboratories (Skin Cancer Center Hornheide, n = 4; Dermatologikum Hamburg, n = 54; IMGM Laboratories Munich, n = 16; CentroDerm Wuppertal, n = 10; all in Germany) using different RNA preparation kits and real-time PCR platforms. Although technical steps are being validated in this analysis, remaining variability due to biological heterogeneity, that is, distinct areas of the same tumor being analyzed, could not be ruled out.

Overall, 93% of replicate determinations confirmed the GEP score (< vs ≥1.3; in-house 100%, externally 94%, 88%, 91%). This demonstrates the robustness of the GEP score regarding technical and biological variability as well as reproducibility in clinical practice regarding varying experimental conditions.

### Clinical Validation of the GEP Score

Our intention was to validate the GEP score under the most stringent clinical conditions. Therefore, a cohort of 211 melanomas was selected in which prognostic assessment by AJCC staging proved to be erroneous. Furthermore, this cohort was selected to be evenly distributed across AJCC stages IA–IIIC (14–32 melanomas/stage) (Table 1) and comprised, in each stage, approximately 40% of melanomas with survival outcomes that differed from those expected according to AJCC staging (ie, early-stage melanomas with MSS of less than five years or late-stage melanomas with MSS of five or more years). The median follow-up in this cohort was 66 (2–316) months.

Also in this cohort of melanomas that were difficult to prognosticate, the dichotomized GEP score discriminated significantly between short-term and long-term survivors (*P* = .002, log-rank test). The independent contribution of the GEP score, AJCC stage, age, and sex was evaluated by multivariable Cox regression analysis (Table 3). The GEP score remained statistically significantly associated with MSS (HR = 1.73, *P* = .0131) in a multivariable analysis accounting for conventional staging. Use of the GEP score correctly reclassified 35% of the patients whose likelihood of surviving five years was misclassified using AJCC staging alone; the use of AJCC staging correctly reclassified 28% of the patients whose likelihood of surviving five years was misclassified using the GEP score alone. In other words, GEP score and AJCC staging when used together in this cohort appeared complementary when predicting the likelihood of death within five years. In a ROC analysis of the validation cohort (Table 4), the synergism of the GEP score and AJCC staging was less obvious, most likely because of the stringent cohort selection criteria outlined above and the resulting weaker performance of AJCC staging.

These data support the prognostic significance of the GEP score above and beyond that of AJCC staging alone in a group of patients whose risk of death within five years was higher than expected using AJCC staging alone.

### Potential AJCC Stage–Dependent Clinical Application of the GEP Score

For AJCC stage–specific evaluation of the correlation of GEP score and clinical outcome, exploratory continuous Cox regression analyses in the combined training and validation cohorts were performed.

**Figure 3.**
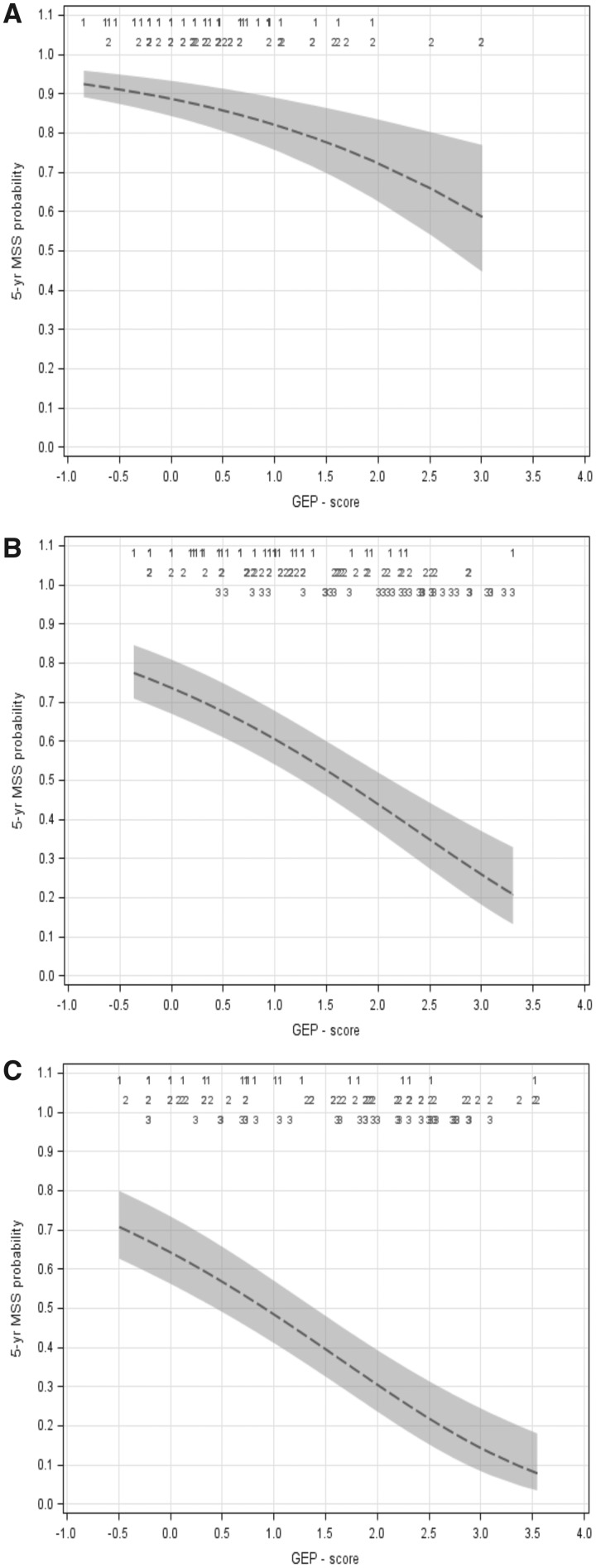
Exploratory continuous Cox regression analysis (in the combined training and validation cohort) of gene expression profiling (GEP) score–dependent melanoma-specific survival (MSS) probability in **(A)** American Joint Committee on Cancer stage I (n = 97), **(B)** stage II (n = 128), and **(C)** stage III (n = 111). The 90% confidence interval and GEP score distribution within each stage, numbered according to substages, are indicated. GEP = gene expression profiling; MSS = melanoma-specific survival.

Whereas AJCC 2009 staging provides single five-year MSS probabilities for stages I (96%), II (75%), and III (55%) (10), the continuous GEP score was inversely correlated to MSS and allowed stage-specific MSS probability prediction across a broad range (stage I: 92%–59%; stage II: 77%–20%; stage III: 71%–8%) (Figure 3). The higher the score, the lower the MSS probability was. The GEP scores in the substages of stages I, II, and III were distributed almost across the entire score range.

In conclusion, the signature-based GEP score complements conventional AJCC melanoma staging by contributing prognostically significant information and refining risk stratification in stages I–III.

## Discussion

Current prognostic assessment of clinical outcome of primary cutaneous melanoma is based on the TNM/AJCC staging system (2). Frequently, however, for patients with similar clinical and histopathological characteristics, outcome varies greatly, ranging from being cured to suffering relapse and death (11). To complement prognostic precision of conventional methods, we have identified a signature of eight genes, whose expression in primary melanoma comprising adjacent stroma correlates with clinical outcome. While expression of one of the signature genes (*KLHL41*) is high in metastatic melanomas (potentially promoting tumor progression), expression of the other seven genes is high in low-risk melanomas (potentially reflecting stromal tumor suppression). However, spatial localization within the tumor area and the potential functional significance of the gene products have not yet been defined.

While three of the signature genes (*KLHL41, KRT9, GBP4*) have not been reported so far to be expressed in melanoma, there is functional evidence for all signature genes suggesting that they might be involved in immune responses, inflammation, or tumor progression. Thus, reduced circulating levels of *DCD* are associated with metastasis of early-stage melanoma (12). Overexpression of mammaglobin 1 (*SCGB2A2*)/lipophilin B (*SCGB1D2*) and *PIP* is linked to good prognosis in ovarian and breast cancer (13,14). Other signature genes are involved in immune responses and inflammation, for example, *KRT9* in the Wnt/β-catenin signaling pathway (15) and *COL6A6* in ostheoarthritis (16); *GBP4* is upregulated by interferon in colon carcinoma, coordinately with the immune checkpoints PD-1/PD-L1 (17). Finally, the risk gene *KLHL41* promotes elongation of pseudopods in transformed cells (18) and hence might stimulate melanoma cell invasion.

Despite efforts to identify biomarkers for melanoma outcome, a prognostic profile providing both scientific quality and clinical validity and utility has not yet been established in clinical routine (19). Prognostic tumor biomarkers are required to meet several important criteria (20,21), such as 1) having been identified in a prospectively collected, unbiased (eg, real-life) training cohort, using an unbiased experimental approach, 2) being technically robust, reproducible, and applicable to FFPE tissue, 3) being based on a predefined, validated algorithm, 4) contributing independent prognostic information, thereby complementing conventional tumor staging, 5) allowing for a clinically significant change in adjuvant tumor treatment.

The first genome-wide prognostic study in melanoma (22) identified a 254-gene classifier expressed in FF primary melanomas, which was associated with progression-free survival in a training cohort of 58 patients (follow-up of four or more years) and a validation cohort of 17 patients. Further immunohistochemical validation in FFPE melanomas revealed that two related genes, MCM4 and MCM6, were associated with patient MSS. However, this study was compromised by relatively small sample cohorts and short clinical follow-up, the inclusion of AJCC stage IV melanomas in the training and validation cohorts, semiquantitative validation lacking a defined prognostic algorithm, and by the lack of demonstration of clinical utility.

Recently, 28 genes have been selected from 71 published melanoma candidate genes and RT-PCR-based gene expression analyzed in FFPE cutaneous melanoma (23). Using machine learning, a signature-based binary risk score was developed, predicting high vs low risk of relapse. However, this study was compromised by the lack of real-life cohorts in score development and validation (24), and cooperativity with AJCC staging has not yet been demonstrated. In addition, application to early melanoma stages is hampered by the relatively high tumor proportion required in the sample (>40%).

Our RT-PCR-based FFPE signature was developed from prognostic candidate genes identified by unbiased whole-genome gene expression profiling in a real-life training cohort of FF melanoma samples containing tumor and adjacent stroma. A signature-based, predefined GEP score was associated with MSS across AJCC stages IA–IIIC, independent of stage and tumor content in the sample. Although the binary GEP score identifies high-risk vs low-risk patient groups, the continuous GEP score provides greater prognostic precision. The GEP score proved to be reproducible and robust by threefold technical validation under different experimental conditions. Stringent clinical validation in melanomas that were difficult to prognosticate by conventional methods confirmed that the association of the GEP score with clinical outcome was statistically significant. The GEP score, as a binary or continuous variable, can be used to stratify clinical outcome within each of the AJCC stages, I, II, and III. We classified our cases according to the seventh edition of the AJCC Cancer Staging Manual (2009), but the eighth edition is now available and includes changes in the classification of melanoma substages (25). We do not expect that these changes in the substaging of melanoma will reduce the ability of the GEP score to discriminate between stages, but this requires independent corroboration.

In summary, the GEP score provides prognostic information complementing AJCC staging. Our data suggest that combining the GEP score with AJCC staging might allow for clinically relevant, stage-specific applications; for example, in stage I, to identify patients at high risk; in stages II/IIIA, risk stratification complementary to prognostic tools such as sentinel lymph node status; and in stage III, identification of high-risk patients in need of adjuvant therapy.

## Funding

Part of this work was supported by the Förderverein Hornheide e.V.

## Notes

Affiliations of authors: Department of Cancer Research (GB, LS) and Department of Dermatology (HJS), Skin Cancer Center Hornheide, Muenster, Germany; Department of Biometry and Clinical Research, Westphalian Wilhelms University, Muenster, Germany (AH); Dermatologikum Hamburg, Hamburg, Germany (TMF, BE, NBS); Department of Medical Oncology, Niels Stensen Clinics, Osnabrück, Germany (JA); Department of Dermatology, University Hospital, Munich, Germany (CB).

The funding source (Fachklinik Hornheide e.V.) was not involved in the design of the study; the collection, analysis, or interpretation of the data; the writing of the manuscript; or the decision to submit the manuscript for publication. GB is the CSO of NeraCare GmbH.

The authors would like to thank Tamara Berger and Maryla Brode for excellent technical assistance.
